# Metformin potentiates anti-tumor effect of resveratrol on pancreatic cancer by down-regulation of VEGF-B signaling pathway

**DOI:** 10.18632/oncotarget.12391

**Published:** 2016-10-01

**Authors:** Mengmeng Zhu, Qiong Zhang, Xiaoling Wang, Licheng Kang, Yinan Yang, Yuansheng Liu, Lei Yang, Jing Li, Liang Yang, Jie Liu, Yin Li, Lingling Zu, Yanna Shen, Zhi Qi

**Affiliations:** ^1^ Department of Histology and Embryology, School of Medicine, Nankai University, Tianjin, China; ^2^ Department of Microbiology, School of Laboratory Medicine, Tianjin Medical University, Tianjin, China; ^3^ Tianjin Institute of Acute Abdominal Diseases of Integrated Traditional Chinese and Western Medicine, Tianjin Nankai Hospital, Tianjin, China; ^4^ Department of Respiratory and Critical Care Medicine, Tianjin Chest Hospital, Tianjin, China; ^5^ Tianjin Key Laboratory of Lung Cancer Metastasis and Tumor Microenviroment, Tianjin Lung Cancer Institute, Tianjin Medical University General Hospital, Tianjin, China

**Keywords:** pancreatic cancer, metformin, resveratrol, VEGF-B, apoptosis

## Abstract

Our previous study showed that resveratrol (RSV) exhibited not only anti-tumor effect, but also had potential tumor promotion effect on pancreatic cancer (Paca) cells through up-regulation of VEGF-B. We determined whether metformin (MET) could potentiate the anti-tumor effect of RSV on PaCa in this study. Combination of RSV (100 μmol/l) and MET (20 mmol/l) significantly inhibited tumor growth and increased apoptosis of human PaCa in comparison with RSV or MET alone treatment in PaCa cell lines (Miapaca-2, Panc-1 and Capan-2). Combination of RSV (60 mg/kg, gavage) and MET (250 mg/kg, i.p.) significantly inhibited tumor growth in PaCa bearing nude mice (subcutaneous injection of 5 × 10^6^ Miapaca-2 cells) in comparison with RSV or MET alone treatment on day 40. Combination treatment significantly decreased VEGF-B expression and inhibited activity of GSK-3β when compared to the RSV alone treatment. Up-regulated expressions of Bax, cleaved caspase-3 and down-regulated expression of Bcl-2 were observed in RSV+ MET group in comparison with RSV group either *in vitro* or *in vivo*. Inhibition of VEGF-B by VEGF-B small interfering RNA (siRNA) mimicked the effects of MET on PaCa cells. These results suggested that MET, a potential pharmacological inhibitor of VEGF-B signaling pathway, potentiated the anti-tumor effect of RSV on PaCa, and combination of MET and RSV would be a promising modality for clinical PaCa therapy.

## INTRODUCTION

Pancreatic cancer (PaCa) is one of the most lethal human cancers with the lowest five-year relative survival rate of only 7% from 2004 to 2010 [1]. Despite major progress in the treatment of PaCa during the last few decades, the survival rate of patients has not significantly improved. In current stage, gemcitabine administration is the standard clinical therapy for the patients with PaCa; however the therapeutic effect was unsatisfactory. The median overall survival time was 5–8 months and one-year survival rate was only 17–25% after gemcitabine alone treatment [2]. Therefore, new treatment options are urgently required.

Vascular endothelial growth factor b (VEGF-B), a member of VEGF family, however, unlike VEGF-A, it is not a major player in angiogenesis [3]. Therefore, VEGF-B has received much less attention for a long time. Recently, Hagberg et al. have found that VEGF-B is a key regulator of endothelial fatty acid uptake [4], and it is a potential target for type 2 diabetes treatments [5]. Since then, VEGF-B signaling pathway gradually became research focus. Its cardioprotective effect [6] and neuroprotective effect [7] have been reported. However, the role of VEGF-B in tumor progress remains controversial. Yang et al. reported that VEGF-B promoted pulmonary metastasis of human melanomas which lead to the poor survival rate of human cancer patients [8]. VEGF-B overexpression predicted for increased distant metastasis and shorter survival in bladder cancer patients [9]. In contrast, Albrecht et al. suggested that over expression of VEGF-B could inhibit pancreatic neuroendocrine tumorigenesis [10].

Resveratrol (trans-3, 4, 5-trihydroxystilbene, RSV), a nonflavonoid polyphenol, is existed in a variety of plant extracts, such as grapes and peanuts [11]. RSV showed multiple biological functions in life science fields. It can protect heart against ischemia-reperfusion injury [12], extend the life span of experimental animal models [13], and ameliorate diabetes-related metabolic changes [14]. Recently, the anti-cancer effect of RSV has been paid much more attention. RSV is shown to prevent a variety of cancers; including hepatocellular carcinoma [15], non-small cell lung adenocarcinoma [16], oral carcinoma [17], osteosarcomas [18], as well as PaCa [19, 20]. Interestingly, in previous study, we found that RSV played dual roles in PaCa cells, namely as a tumor suppressor via the promotion of apoptosis; as a tumor activator via the up-regulation of VEGF-B; and the anti-cancer effect of RSV was much stronger than the cancer promotion effect [21]. Therefore, we assumed that combination of RSV with pharmacological inhibitor of VEGF-B signaling pathway could potentiate the anti-tumor effect of RSV, and would be a promising modality for clinical PaCa therapy.

Metformin (MET) has been widely used as a first-line anti-diabetic medicine for type 2 diabetes. It effectively decreases blood glucose level mainly through the inhibition of hepatic glucose secretion [22]. MET potentiated rapamycin and cisplatin effects on gastric cancer in mice [23] and induced ER stress-dependent apoptosis through miR-708-5p/NNAT pathway in prostate cancer [24]. Interestingly, MET decreased the incidence of PaCa in patients with diabetes, and this effect was not related to the blood glucose level of patients [25]. However, the underlying mechanisms of MET for its anti-cancer effect are not fully understood.

To our knowledge, the combination of RSV and MET has not ever been applied in the treatment of PaCa before. We aimed to examine whether MET could potentiate the anti-tumor effect of RSV on PaCa cells, and whether this effect was related to the down-regulation of VEGF-B signaling pathway.

## RESULTS

### Combination of RSV and MET dramatically inhibits survival of human PaCa cells

**Figure 1:**
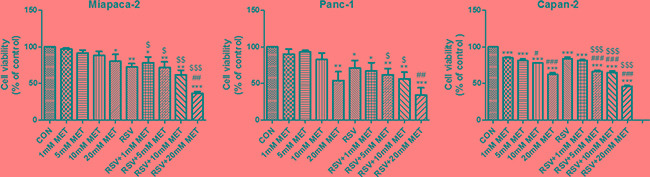
Effect of RSV and MET alone or combination treatment on viability of PaCa cells CCK8 assay was performed to examine the survival of Miapaca-2, Panc-1 and Capan-2 cells after 48 h of drugs treatment. Graphs represent mean ± SEM (*n* = 3). ^*^p < 0.05, ^**^p < 0.01, ^***^p < 0.001 vs. CON group; ^#^*p* < 0.05, ^##^*p* < 0.01, ^###^*p* < 0.001 vs. RSV group; ^$^*p* < 0.05, ^$$^*p* < 0.01, ^$$$^*p* < 0.001 vs. MET group.

To explore the effect of RSV and MET on the survival of PaCa cells, CCK8 assay was performed after 48 h of drugs treatment. RSV alone treatment significantly decreased viability of human PaCa cells in comparison with control (CON) group. No significant differences of cell viability were found between CON group and MET low concentration (1, 5 or 10 mM) alone treatment group in Miapaca-2 and Panc-1 cells. In contrast, cell viability was significantly decreased after treatment with 20 mM MET alone in all three cell lines. Combination of RSV and MET at different concentrations significantly inhibited PaCa cells survival when compared to the CON group. Noticeably, the combination of RSV and 20 mM MET dramatically decreased cell viability to 36.61 ± 2.45%, 34.06 ± 10.01%, and 46.06 ± 2.1% in Miapaca-2, Panc-1, and Capan-2 cells, respectively. Furthermore, RSV+20 mM MET group showed significant lower cell viability in comparison with RSV alone group or 20 mM MET alone group in three cell lines, indicating the synergistic effect of 100 μM RSV and 20 mM MET on PaCa cells (Figure 1). Therefore, we selected the concentration of 20 mM of MET to serve as the working concentration in the subsequent studies.

### Combination of RSV and MET inhibits VEGF-B signaling pathway in comparison with RSV alone treatment in human PaCa cells

**Figure 2:**
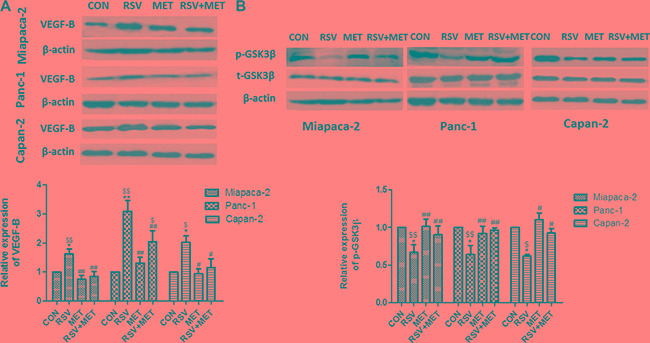
Effect of RSV and MET alone or combination treatment on VEGF-B signaling pathway in PaCa cells Western blot and quantification of VEGF-B (**A**), and phosphorylated GSK-3β (**B**) in three PaCa cell lines. Graphs represent mean ± SEM (*n* = 5).*p < 0.05 vs. CON group; ^#^ *p* < 0.05, ^##^*p* < 0.01 vs. RSV group; ^$^*p* < 0.05, ^$$^*p* < 0.01 vs. MET group.

To investigate the relationship between the drug treatments and VEGF-B signaling pathway, western blots for VEGF-B and GSK-3β were performed (Figure 2). RSV alone treatment significantly elevated the protein level of VEGF-B to approximately 1.5-fold, 3-fold and 2-fold in Miapaca-2, Panc-1, and Capan-2 cells, respectively. MET alone treatment did not directly decrease the VEGF-B expression in comparison with CON group in three PaCa cells. However, the combination treatment significantly lowered VEGF-B protein level when compared to RSV alone treatment in all three cell lines, indicating that MET could attenuate RSV- inducing up-regulation of VEGF-B in PaCa cells (Figure 2A).

Furthermore, we examined the protein level of GSK-3β, which has been reported as a down-stream molecule of VEGF-B in our previous study [21]. GSK-3β served as a tumor activator in pancreatic cancer, and phosphorylation at Ser 9 reduced the activity of this kinase [26]. RSV alone treatment markedly decreased the phosphorylation of GSK-3β at Ser 9, indicating that RSV activated the GSK-3β and served as a potential tumor activator in pancreatic cancer. MET alone treatment did not change the protein level of phosphorylated GSK-3β in comparison with CON group. Whereas the protein level of phosphorylated GSK-3β in RSV+ MET group was significantly higher than those in RSV group (Figure 2B).

### MET potentiates the apoptotic effect of RSV, and inhibition of VEGF-B mimicked the effect of MET on human PaCa cells

**Figure 3:**
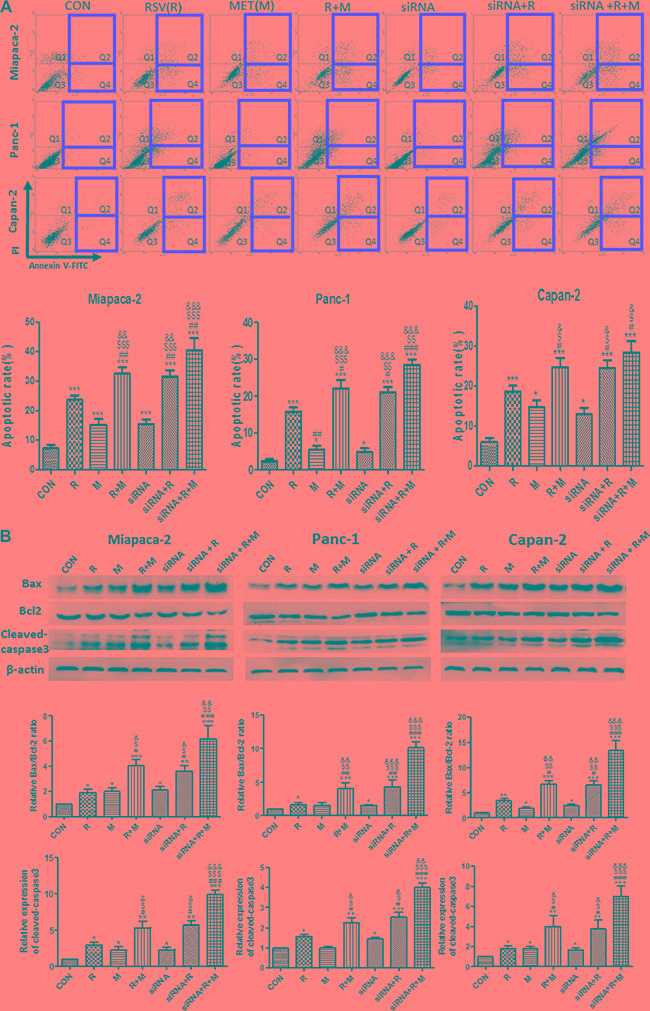
Effect of RSV, MET and VEGF-B siRNA alone or combination treatment on apoptosis of PaCa cells Flow cytometry test was performed to determine the apoptotic effect of single or combined treatment on PaCa cells (**A**). Western blot and quantification of the protein level for Bax, Bcl-2 and cleaved-caspase3 (**B**) was represented. Graphs represent mean ± SEM (*n* = 3). ^*^p < 0.05, ^**^p < 0.01, ^***^p < 0.001 vs. CON group; ^#^*p* < 0.05, ^##^*p* < 0.01, ^##^#*p* < 0.001 vs. RSV group; ^$^*p* < 0.05, ^$$^*p* < 0.01, ^$$$^*p* < 0.001 vs. MET group; ^&^*p* < 0.05, ^&&^*p* < 0.01, ^&&&^*p* < 0.001 vs. siRNA group.

To evaluate the apoptotic effects of drug treatments and confirm the role of VEGF-B in tumor apoptosis, flow cytometry analysis and VEGF-B small interfering RNA (siRNA) treatment were carried out *in vitro.* The inhibitory efficiencies of VEGF-B siRNA were shown in Supplementary Figure S1. RSV or MET alone treatment significantly increased apoptotic rate in comparison with CON group. Apoptotic rate in combination group was higher than those in RSV and MET alone treatment group in all three cell lines, indicating that MET potentiated the apoptotic effect of RSV on human PaCa cells. VEGF-B siRNA alone treatment increased apoptosis in all three kinds of PaCa cells in comparison with CON group. Combination of VEGF-B siRNA and RSV showed higher apoptotic rate when compared to RSV, MET or siRNA alone treatment group in all three cell lines. No significant difference was observed between RSV+MET group and siRNA+RSV group (Figure 3A).

Protein levels of Bax, Bcl-2 and cleaved caspase3 were analyzed by western blot. Similar with the tendency of apoptotic rate in Figure 3A, RSV+MET group showed higher expressions of Bax and cleaved caspase3, and lower expression of Bcl-2 than RSV or MET alone treatment group. siRNA+RSV group showed higher ratio of Bax/Blcl-2 and expression of cleaved caspase3 than RSV or siRNA alone treatment group in all three cell lines. Moreover, there was no significant differences in protein levels of Bax, Bcl-2 and cleaved caspase 3 between RSV+MET group and siRNA+RSV group in all three cell lines (Figure 3B).

### Combination of RSV and MET inhibits tumor growth in the xenografts model

**Figure 4:**
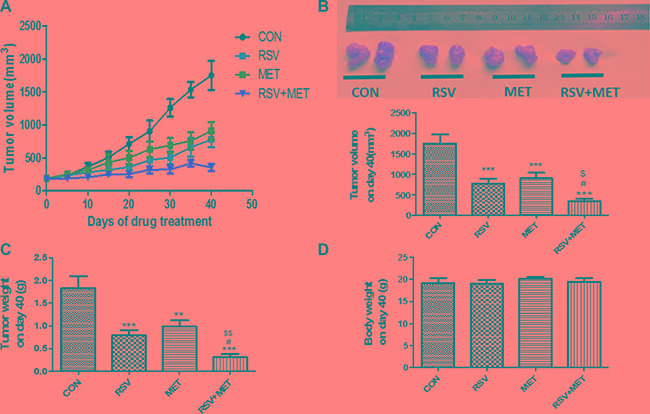
Effect of RSV and MET alone or combination treatment on tumor growth in xenografts model Miapaca-2 cells were injected subcutaneously into the flanks of nude mice. Drug administration was maintained for 40 days, and then mice were sacrificed. Tumor growth curves (**A**), tumor volume (**B**), tumor weight (**C**) and mice body weight (**D**) on day 40 were presented. Graphs represent mean ± SEM (CON group: *n* = 6, other three groups: *n* = 8). ^*^p < 0.05, ^**^p < 0.01, ^***^p < 0.001 vs. CON group; ^#^*p* < 0.05, ^##^*p* < 0.01, ^###^*p* < 0.001 vs. RSV group; ^$^*p* < 0.05, ^$$^*p* < 0.01, ^$$$^*p* < 0.001 vs. MET group.

To further investigate the anti-tumor effects of the RSV and MET combination treatment, Miapaca-2 cells were injected into subcutaneous site of nude mice. Either RSV or MET alone treatment markedly inhibited tumor growth, and combination treatment was much more effective than their alone treatments in tumor volumes (Figure 4A). RSV+ MET group also showed the smallest area of under curves (AUC) among four groups (data not shown). RSV or MET alone treatment significantly decreased the tumor volume and tumor weight in comparison with CON group on day 40 (Figure 4B, 4C). Meanwhile, RSV+MET group showed the smallest tumor volume (Figure 4B) and the lightest tumor weight (Figure 4C) in the four groups on day 40. No significant differences on nude mice body weight were observed among four groups (Figure 4D).

**Figure 5:**
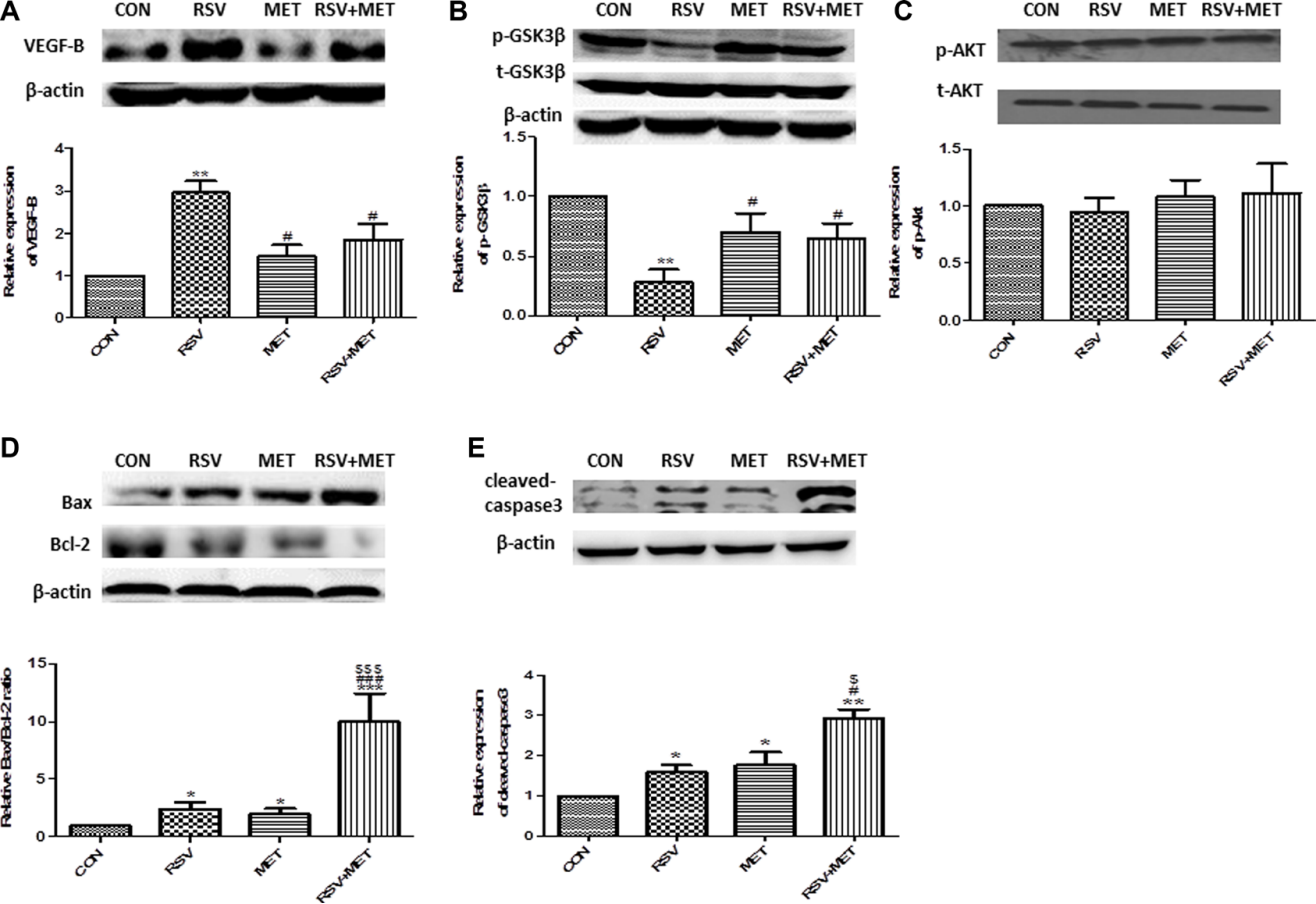
Effect of RSV and MET alone or combination treatment on VEGF-B signaling pathway and tumor apoptosis in xenografts model The expression of VEGF-B (**A**), phosphorylated GSK-3β (**B**), phosphorylated Akt (**C**), Bax and Bcl-2 (**D**), and cleaved-caspase3 (**E**) were presented. Graphs represent mean ± SEM (CON group: *n* = 6, other three groups: *n* = 8). ^*^p < 0.05, ^**^p < 0.01 vs. CON group; ^#^*p* < 0.05 vs. RSV group; ^$^*p* < 0.05 vs. MET group.

Consistent with the results *in vitro*, we found that VEGF-B expression was up-regulated after RSV alone treatment, whereas, combination of RSV and MET significantly attenuated this up-regulation of VEGF-B (Figure 5A). Meanwhile, RSV+MET treatment increased phosphorylation of GSK-3β at Ser 9 (Figure 5B). Contrarily, combination treatment did not change the expression of phosphorylated Akt at S473 (Figure 5C). Furthermore, combination treatment resulted in the highest ratio of Bax/Bcl-2 and expression of cleaved caspase 3 in four groups, indicating that MET potentiates the apoptotic effect of RSV on PaCa xenograft model (Figure 5D, 5E).

### Histological assessment in mice xenograft model

**Figure 6:**
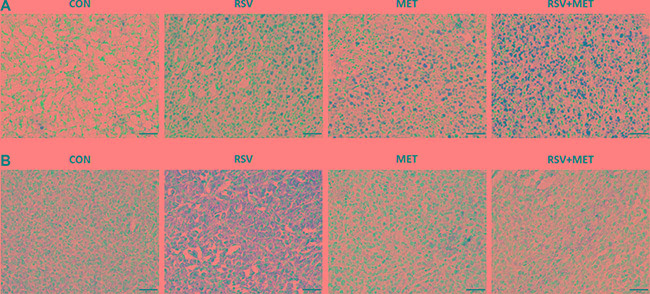
Histological assessment in mice xenograft model To further determine the apoptotic effect of RSV and MET combination treatment, TUNEL staining was performed in tumor tissues (**A**). Meanwhile, immunohistochemical staining for VEGF-B was also performed in tumor tissues (**B**). The black line represented a scale bar of 50 μm.

Terminal deoxynucleotidyl transferase-mediated nick end labeling (TUNEL) staining was performed in tumor tissues to further determine the apoptotic effect of combination treatment. Few TUNEL positive cells were observed in CON group, whereas, more TUNEL positive cells (the nuclei were stained with brown) were found either in RSV or MET group. Remarkably, abundant TUNEL positive cells were observed in RSV+ MET group (Figure 6A).

Immunohistochemical staining of VEGF-B was also carried out in the xenograft tissues. As shown in Figure 6B, increased VEGF-B positive areas (stained with brown) were observed in RSV group in comparison with CON group, whereas, combination treatment obviously inhibited VEGF-B expression in tumor tissues, indicating that combination of RSV and MET do down-regulated the expression of VEGF-B in comparison with RSV alone treatment.

### Schematic model of this study

**Figure 7:**
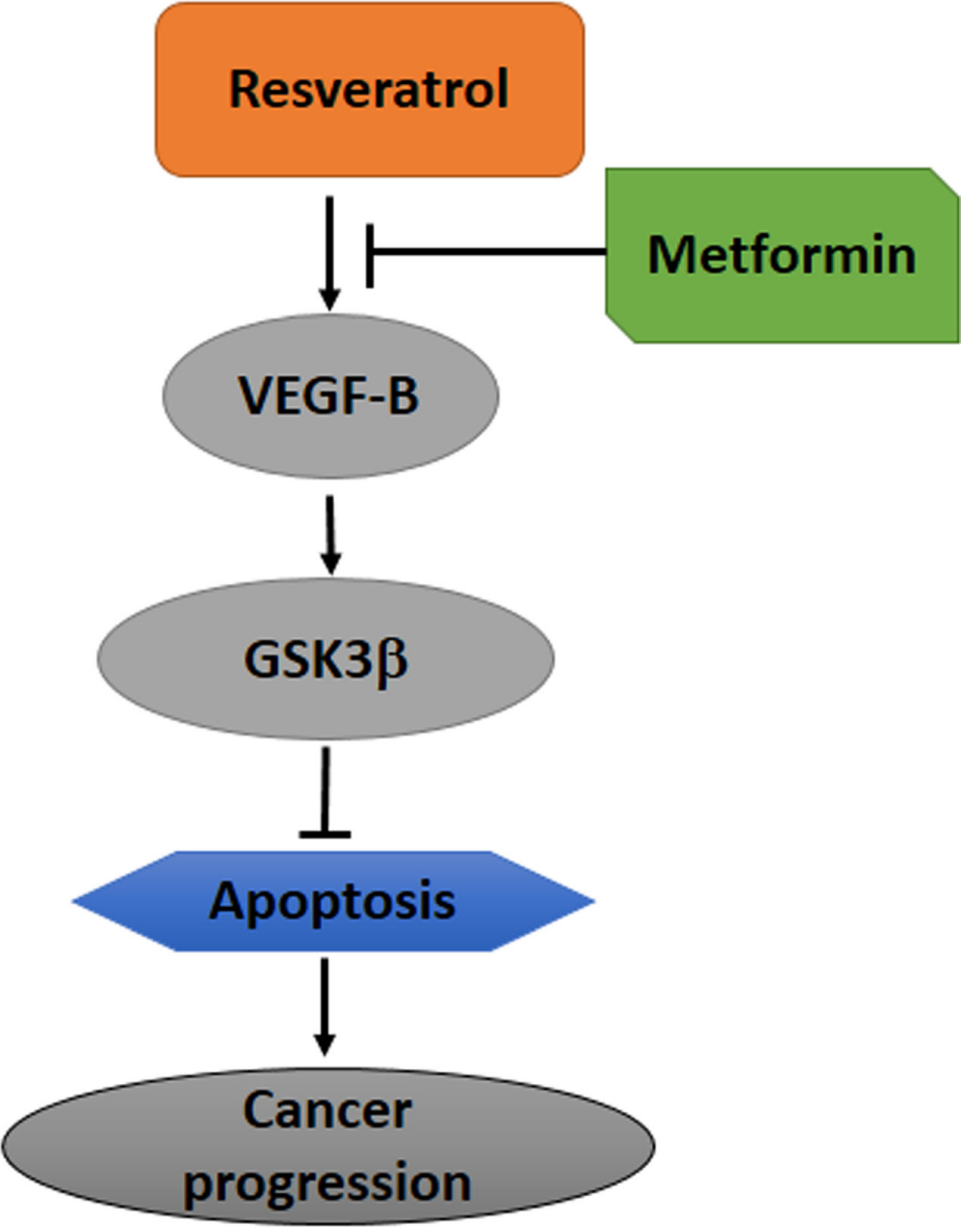
Schematic model of this study MET potentiates the anti-cancer effect of RSV via inhibition of VEGF-B/GSK-3β signaling pathway.

Taken together with all results, we can conclude that MET potentiates the anti-cancer effect of RSV via inhibition of VEGF-B/GSK-3β signaling pathway, then promotes apoptotic effect of RSV to prevent pancreatic cancer progression (Figure 7). Therefore, the combination of RSV and MET can be used as a promising approach in clinical pancreatic cancer therapy.

## DISCUSSION

Previous study showed that RSV inhibited apoptosis of PaCa cells via up-regulating expression of VEGF-B [21]. Therefore, we assumed that combination of RSV and a pharmacological inhibitor of VEGF-B signaling pathway should potentiate the anti-tumor effect of RSV on pancreatic cancer. However, the approaches for the inhibition of VEGF-B are limited to the specific neutralizing anti-VEGF-B antibody [5], siRNA [21], and the genetic knock out animals [4, 5, 27]. Anti-VEGF-B antibody is usually used *in vivo* study and siRNA is limited to the *in vitro* study. These two approaches cannot be applied in clinical study in current stage. To our knowledge, pharmacological inhibitor of VEGF-B signaling pathway has not been found yet. Discovering new drugs for inhibition of VEGF-B signaling pathway therefore remains an urgent priority in the cancer therapy.

MET has been proven to inhibit tumor growth [28–30]. However, the relationship between MET and VEGF-B signaling pathway has not been reported yet. In our other preliminary study, we found that MET down-regulated the expression of VEGF-B in rat cardiomyocytes *in vitro* (data not shown). Moreover, Cheng et al. found that serum VEGF-B was elevated in women with polycystic ovary syndrome and can be decreased with metformin treatment [31]. MET has been widely used in clinical therapy with low side effects, therefore the clinical application prospect of MET is much better than the neutralizing antibody and siRNA treatments. Naturally, we assumed that MET might down-regulate the expression of VEGF-B in PaCa, therefore could increase the anti-tumor effect of RSV in PaCa.

Both RSV [32, 33] and MET [34, 35] can induce apoptosis in various type of cancer cells. We found that RSV and MET alone treatment significantly increased apoptosis in comparison with CON group either *in vitro* or *in vivo.* Notably, combination group showed the highest apoptotic rate, ratio of Bax/Bcl-2 and expression of cleaved-caspase 3 with the most TUNEL positive cells in four groups (Figures 3, 5D, 5E, 6A), indicating that combination treatment resulted in an enhancement of their *in vitro* and *in vivo* apoptotic effect.

In our previous study, we found that GSK-3β activity had significantly increased after RSV treatment, and its activity had significantly decreased after inhibition of VEGF-B by VEGF-B siRNA, indicating that GSK-3β was the down-stream of VEGF-B [21]. MET did not directly decrease the expression of VEGF-B in comparison with CON group, combination treatment markedly down-regulated the expressions of VEGF-B and decreased the activity of GSK-3β in comparison with RSV group (Figures 2, 5A, 5B), revealing that MET attenuates RSV-inducing activation of VEGF-B signaling pathway. Although the mechanism was not fully understood, MET may exert this effect via the regulation of some molecule which locates in the up-stream of VEGF-B.

Some studies showed that phosphorylation of Akt (S473) could regulate the activity of GSK-3β [36, 37]. We examined phosphorylation of Akt after drugs treatment. Similar with Ku's results [38], we found that although RSV and combination treatment changed the expression of phosphorylated GSK-3β, phosphorylation of Akt (S473) was not altered (Figure 5B–5C), suggesting that Akt did not regulate activity of GSK-3β in this study.

We inhibited VEGF-B expression by using siRNA to confirm the inhibitory effect of VEGF-B on apoptosis. VEGF-B siRNA alone treatment increased apoptosis when compared to the CON group. Combination of siRNA and RSV showed higher apoptotic rate than RSV alone treatment. Interestingly, there is no significant difference in tumor apoptosis between RSV+siRNA group and RSV+MET group (Figure 3), indicating that MET increased apoptosis due to inhibition of VEGF-B signaling pathway.

Taken together, our results indicate that the treatment of PaCa with combinations of RSV and MET can be more effective in inhibiting tumor cell growth than the treatment with RSV or MET alone either *in vitro* or *in vivo*, and the molecular mechanism was related to the change of VEGF-B expression. To our knowledge, this is the first time that the combination of RSV and MET was applied in the treatment of PaCa. Although the additional studies should be performed further to fully define the therapeutic potential of these compounds, this combination will be a promising approach for clinical pancreatic cancer therapy.

## MATERIALS AND METHODS

### Reagents and antibodies

Resveratrol (for *in vitro* experiments) and metformin were purchased from Sigma-Aldrich (St. Louis, USA). Resveratrol (for *in vivo* experiment) was purchased from DND Pharm-Technology Corporation (Shanghai, China). Cell counting kit-8 (CCK-8) was purchased from Dojindo Laboratories (Kumamoto, Japan). FITC annexin V apoptosis detection kit was purchased from BD (CA, USA). The anti- VEGF-B antibody was obtained from Abcam (MA, USA). Antibodies against Akt, phosphorylated Akt (Ser473), GSK3β, phosphor-GSK3β (Ser9), cleaved-caspase3 were obtained from Cell Signaling Technology (MA, USA). Antibodies to Bax, Bcl-2 and β-actin were purchased from Santa Cruz Biotechnology (CA, USA).

### Animals

Male nude mice (nu/nu, 6 weeks old) were purchased from Beijing Military Academy of Medical Sciences Laboratory Animal Center, Beijing, China. All mice were housed under specific pathogen free conditions with a controlled temperature (25°C) in a 12 h light/dark cycle. Mice were randomized into four groups (*n* = 6–8): no treatment group (CON); resveratrol administration group (RSV); metformin administration group (MET); combination treatment group (RSV + MET). The approval to conduct this experiment was obtained from the Animal Care Committee of School of Medicine, Nankai University, and all animals were treated according to the experimental protocols under its regulations.

### Cell lines and culture conditions

The PaCa cell lines, Miapaca-2 (ATCC CRL-1420), Panc-1(ATCC CRL-1469), and Capan-2(ATCC HTB-80) were purchased from the Type Culture Collection of the Chinese Academy of Sciences, Shanghai, China. Cells were cultured in DMEM medium supplemented with 10% fetal bovine serum (FBS), 100 U/ml penicillin and 100 μg/ml streptomycin n in 95% air and 5% CO2 at 37°C. Culture medium was changed every 2 days, and cells were subcultured once they reached 70–80% confluence. Cells were plated at an appropriate density according to each experimental design.

### CCK-8 cell proliferation assay

CCK8 assay was performed as described previously [21]. In brief, PaCa cells were plated into 96-well plates, then treated with MET (1, 5, 10, 20 mM), RSV (100 μM) alone, or combination of two drugs for 48 h. The optimal concentration of RSV was determined to 100 μM according to the results of previous study [21]. At the end of the incubation, CCK8 working solutions were added to each well and incubated for 2 h at 37°C, then measured absorbance value at 450 nm with a microplate reader (Thermo, China). Cell viability was calculated by the ratio of the absorbance in treatment group to the absorbance in untreated group. Untreated cells were used as the control (CON).

### Flow cytometry analysis

Apoptosis of PaCa cells was quantified by flow cytometry after cells were stained with Annexin V and propidium iodide (PI) [21]. Briefly, cells were washed twice with PBS, and then 5 μl of AnnexinV and 5 μl of PI were added into 100 μl cell suspension (1 × 10^5^ cells) and incubated for 15 min at room temperature. Apoptotic cells were defined as the Annexin V positive cells, namely, the sum of the early apoptotic cells (quadrant 4, Q4) and late apoptotic cells (quadrant 2, Q2). The apoptotic rate was defined as the ratio of the number of apoptotic cells / the number of all cells. The assay was then performed, and the data were analyzed WinMDI 2.9 software.

### Western blot analysis

Western blot was done as previously described [39]. In brief, cells were lysed and incubated for 30 min in RIPA buffer. Protein concentration was measured using BCA protein assay Kit (Thermo, USA). The proteins were electroblotted to a polyvinylidene fluoride (PVDF) membrane (Millipore, USA). The membrane was incubated with a primary antibody against VEGF-B, Akt, phosphorylated Akt, GSK3β, phosphorylated GSK3β, Bax, Bcl-2, cleaved-caspase3 and β-actin.

### Tumor xenograft

Human PaCa cell line, Miapaca-2 cells were used to make the tumor xenograft model in nude mice. Briefly, 5 × 10^6^ cells were suspended in 100 μL PBS, and then cells were injected subcutaneously into the flanks of mice. Drug treatment was initiated 10 days after injection of the cells. No treatment was performed in CON group. Mice in RSV group received daily resveratrol administration (60 mg/kg, gavage injection), and mice in MET group received daily metformin administration (250 mg/kg, i.p.). Both gavage injection of RSV and i.p. injection of MET were performed in RSV+MET group. Drug administration was maintained for 40 days, and then mice were sacrificed. Body weight, tumor weight and tumor volume were monitored throughout the experiment. The length, width and depth of the tumors were measured using calipers every 3 days. The tumor volume was calculated as V = 0.52× (length × width × depth).

### TUNEL staining

Mice tumor tissues were fixed in 4% paraformaldehyde solution for 24h, followed by immersion in 70% ethanol at 4°C for 24–48 h. Then, Next, the samples were embedded in paraffin and 5 μm thick sequential sections were cut. TUNEL staining was performed according to the protocol of TUNEL Staining Kit (Roche, USA) to determine the apoptosis of cancer cells. The nuclei of apoptotic cells were stained with brown.

### Immunohistochemical staining

Immunohistochemical staining for VEGF-B was carried out. The main staining protocol was described previously [40]. In brief, tissue sections were washed in dH_2_O, and then blocked with 10% normal goat serum for 30 minutes. Subsequently, primary antibody (VEGF-B) was applied overnight at 4°C. Thereafter, they were incubated with peroxidase conjugated second antibody (goat anti-rabbit immunoglobulins, Promega, China) diluted to 1:100 in PBS for 30 min. After washing in PBS, coloring reaction was carried out.

### VEGF-B siRNA transfection

VEGF-B siRNA transfection was performed as previously described [21]. Briefly, Paca cells were cultured in RPMI-1640 medium (FBS free) for 2 h before siRNA transfection. All siRNA were purchased from GenePharma (shanghai, China). VEGF-B siRNA (100 nM), or negative control (NC) siRNA was mixed with lipofectamine 2000 (Invitrogen, USA). The cells were incubated with the transfection mixture for 6 h and then washed with RPMI-1640 medium containing 10% FBS. The cells were incubated for an additional 24 h before harvest.

### Statistical analysis

All experiments were repeated three times, and all results were expressed as the mean ± SEM. Significant differences between multiple groups were tested by one-way analysis of variance (ANOVA) followed by multiple comparisons performed with LSD test (SPSS ver. 17). Statistical significance was defined as *p* < 0.05.

## SUPPLEMENTARY MATERIALS FIGURES AND TABLES


