# Supplementary material for: Metformin potentiates anti-tumor effect of resveratrol on pancreatic cancer by down-regulation of VEGF-B signaling pathway
Source: Oncotarget. 2016 Oct 1;7(51):84190–200. doi: 10.18632/oncotarget.12391 (PMC5356654; doi:10.18632/oncotarget.12391)
Supplement: Supplementary file 1 [file oncotarget-07-84190-s001.pdf]

# Metformin potentiates anti-tumor effect of resveratrol on pancreatic cancer by down-regulation of VEGF-B signaling pathway

## Supplementary Materials

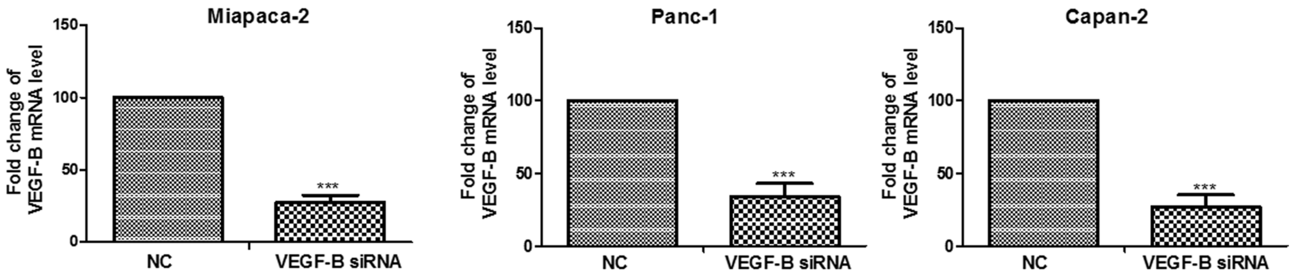


**Supplementary Figure S1: The inhibitory efficiencies of VEGF-B siRNA in PaCa cells.** VEGF-B siRNA treatment significantly decreased mRNA levels of VEGF-B in comparison with negative control siRNA (NC) in three PaCa cells.
